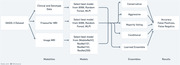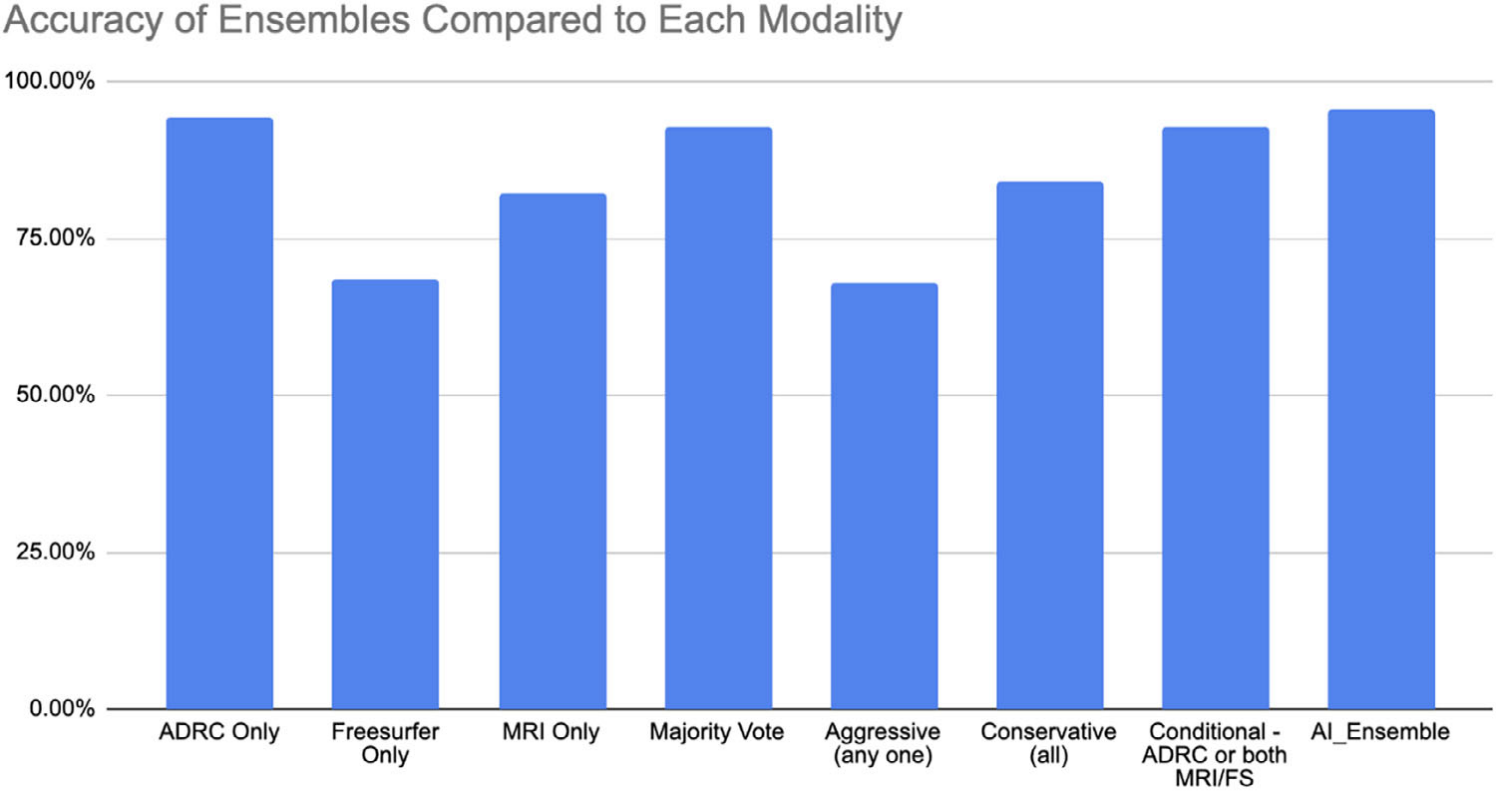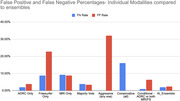# Machine Learning Ensemble Prediction of Clinical Dementia Rating Using MultiModal Data

**DOI:** 10.1002/alz.089241

**Published:** 2025-01-03

**Authors:** Danika Gupta, Sindhu Ghanta

**Affiliations:** ^1^ The Harker School, Saratoga, CA USA; ^2^ Pyxeda, Foster City, CA USA

## Abstract

**Background:**

Early detection and accurate forecasting of AD progression are crucial for timely intervention and management. This study leverages multi‐modal data, including MRI scans, brain volumetrics, and clinical notes, utilizing Machine Learning (ML), Deep Learning (DL) and a range of ensemble methods to enhance the forecasting accuracy of Alzheimer’s disease.

**Method:**

We utilize the OASIS‐3 longitudinal dataset, tracking 1,098 patients over 30 years. From OASIS‐3, we combined three modalities ‐ MRI scans, Freesurfer brain volumetrics, and Clinical Data from the Alzheimer’s Disease Research Center (ADRC). We use Convolutional Neural Networks (CNNs), specifically MobileNetV2, ResNet101, ResNet152 and ResNet200 for MRIs and Machine Learning (ML) techniques (Random Forest and K Nearest Neighbors) for Freesurfer featurized brain volumetrics and clinical data. Individual models were tuned for each modality, with the best models combined via ensembles to predict each patient’s future Clinical Dementia Rating (CDR). Ensembles evaluated included: aggressive (any modality predicting positive), conservative (all modalities predicting positive), conditional ensembles (majority voting and ADRC or both MRI/Freesurfer), and custom machine learning models built to integrate the modality predictions based on the confidence values returned from the MRI model and the predictions of other models. The figure below shows our experimental pipeline.

**Result:**

The study achieved >95% accuracy in predicting future CDR. Ensembles notably reduced harmful False Negatives by 2x‐15x, compared to individual modalities, while incurring nominal increases in False Positives. The machine learning trained ensemble demonstrated improved accuracy over the best individual modality predictions The results highlight the potential of multi‐modal AI ensemble methods in improving the accuracy of early AD detection and prognosis. The figures below show comparative accuracy and false positives/false negatives rates for each ensemble as compared to the individual modalities.

**Conclusion:**

This work demonstrates the potential efficacy of multi‐modal data integration via ensemble learning in forecasting Alzheimer’s disease, significantly outperforming single‐modality methods. It underscores the importance of leveraging diverse data sources and advanced analytical techniques for early diagnosis and intervention in Alzheimer’s care, paving the way for future research to explore additional modalities and methods for even greater accuracy and clinical utility.